# Design and validation of a PCR screen for γ-butyrolactone-like regulatory systems in *Streptomyces*

**DOI:** 10.1099/acmi.0.000661.v3

**Published:** 2023-09-12

**Authors:** Valentin Waschulin, Chiara Borsetto, Christophe Corre, Elizabeth M. Wellington

**Affiliations:** ^1^​ School of Life Sciences, University of Warwick, Coventry CV4 7AL, UK; ^2^​ Department of Chemistry, University of Warwick, Coventry CV4 7AL, UK

**Keywords:** autoregulators, butenolides, furans, secondary metabolism, specialised metabolism, *Streptomyces*, γ-butyrolactones

## Abstract

γ-butyrolactone and related signalling systems are found in *Streptomyces* and other actinobacteria where they control the production of secondary or specialized metabolites such as antibiotics. Genetic manipulation of these regulatory systems therefore leads to changes in the secondary metabolite profile of a strain and has been used to activate previously silent secondary metabolite gene clusters. However, there is no easy way to assess the presence of γ-butyrolactone-like systems in *Streptomyces* strains without whole-genome sequencing. We have therefore developed and tested a PCR screen that is able to detect homologues of the commonly co-located butenolide synthase and γ-butyrolactone receptor genes. This PCR screen could be employed for the screening of strain libraries to detect signalling systems without the necessity for whole-genome sequencing.

## Data Summary

Sanger sequencing reads and the genome sequences of the three Antarctic *Streptomyces* isolates are available in the zenodo repository under the DOI 10.5281/zenodo.8272490.

## Introduction

γ-butyrolactones and related small molecules such as methylenomycin furans and butenolides are autoregulatory molecules involved in the control of the life cycle and secondary metabolite production in many *Streptomyces* species as well as other Actinobacteria. Their mechanism of action involves the biosynthesis of an autoregulator molecule, which then binds to a cognate receptor protein, which acts as a transcriptional repressor. The binding sites of these repressors are known as autoregulatory response elements (AREs) and contain palindromic sequences that are strongly conserved within a regulatory system. Through autoregulator binding, the receptor protein dissociates from the ARE and thereby allows for transcription of the downstream genes. This sets in motion a regulatory cascade, resulting in changes in primary metabolism and/or changes in secondary metabolite production [1–3]. The effects are often pathway-specific, but can also be pleiotropic, affecting many different processes. Manipulations of autoregulatory systems have been successfully employed to elicit the production of novel secondary metabolites in *Streptomyces* [4, 5]. While multiple layers of regulation as well as knock-on effects can make the prediction of the outcome of such manipulations difficult, changes in the secondary metabolite profile are almost always observed. This is especially true for deletions of the pseudoreceptor, a second TetR-like repressor often involved in the regulatory cascade [6–11]. This makes these autoregulatory systems useful molecular tools for secondary metabolite discovery. In many cases, it has furthermore been observed that autoregulator biosynthesis and receptor genes are collocated on the chromosome. This collocation makes it easier to identify and eventually manipulate the components of the system in order to elicit changes in the secondary metabolite profile of the strain. Currently, however, whole-genome sequencing is necessary to detect the presence of γ-butyrolactone-related genes or to determine the collocation of genes. While sequencing costs have been falling for a long time, *Streptomyces* genomes are notoriously difficult to assemble, often requiring long reads for contiguous assembly [12]. Therefore, a PCR screen could help detect autoregulatory systems without the need to sequence the strain beforehand. In the present work, we designed a PCR assay for detecting collocated autoregulator biosynthesis and receptor genes. This assay could allow the screening of culture collections for potentially manipulable autoregulatory systems without whole-genome sequencing.

## Methods

### Genome download, cblaster searches and gene orientation

All available *Streptomyces* assemblies were obtained from GenBank using ncbi-genome-download and gimme-taxa.py [13], resulting in 2040 downloaded assemblies. A cblaster v1.3.13 [14] database was constructed and searches for co-occurrences of ScbA and ScbR homologues were carried out using cblaster (E-value <0.02) with ScbR (CAB60184.1) and ScbA (CAB60185.1) as input. The occurrences of different gene orientations (i.e. the location of one or both genes on forward or reverse strand) in the output were counted using R packages *dplyr* and *stringr*.

### Alignment and sequence logo visualization

The protein sequences of the ScbA and ScbR homologues in the 1092 hits were aligned using clustalo 1.2.4 [15] and a logo was visualized using skylign.org [16] to visualize conserved amino acids to serve as degenerate primer sites. Primers were designed with the most conserved motif as the 3′ end and using a *Streptomyces coelicolor* codon table [17]. Primers were purchased from IDT.

### Motif count

To estimate the likely efficiency of the primers, the amino acid sequences of the divergently oriented ScbA/ScbR homologues were extracted and the co-occurrence of different binding-site variations was analysed using seqkit 2.2.0 [18].

### PCR

PCR was conducted on 50 ng of genomic DNA using KAPA Taq polymerase (Sigma-Aldrich) with each 25 μl reaction containing 1 μl BSA (2%), 1.25 μl DMSO, 2.5 μl KAPA Taq buffer, 0.5 μl dNTPs (10 mM), 2 μl of each primer (10 μM), and 0.1 μl polymerase, at 55 °C with 45 s extension time and 35 cycles. Bands were visualized on 1.5 % agarose gels using GelRed.

### ARE motif analysis

The Sanger sequences were submitted to meme v.5.5.3 [19] using classic mode with a minimum width of 15 and a maximum width of 30 nucleotides.

## Results

### Primer design

**Fig. 1.**
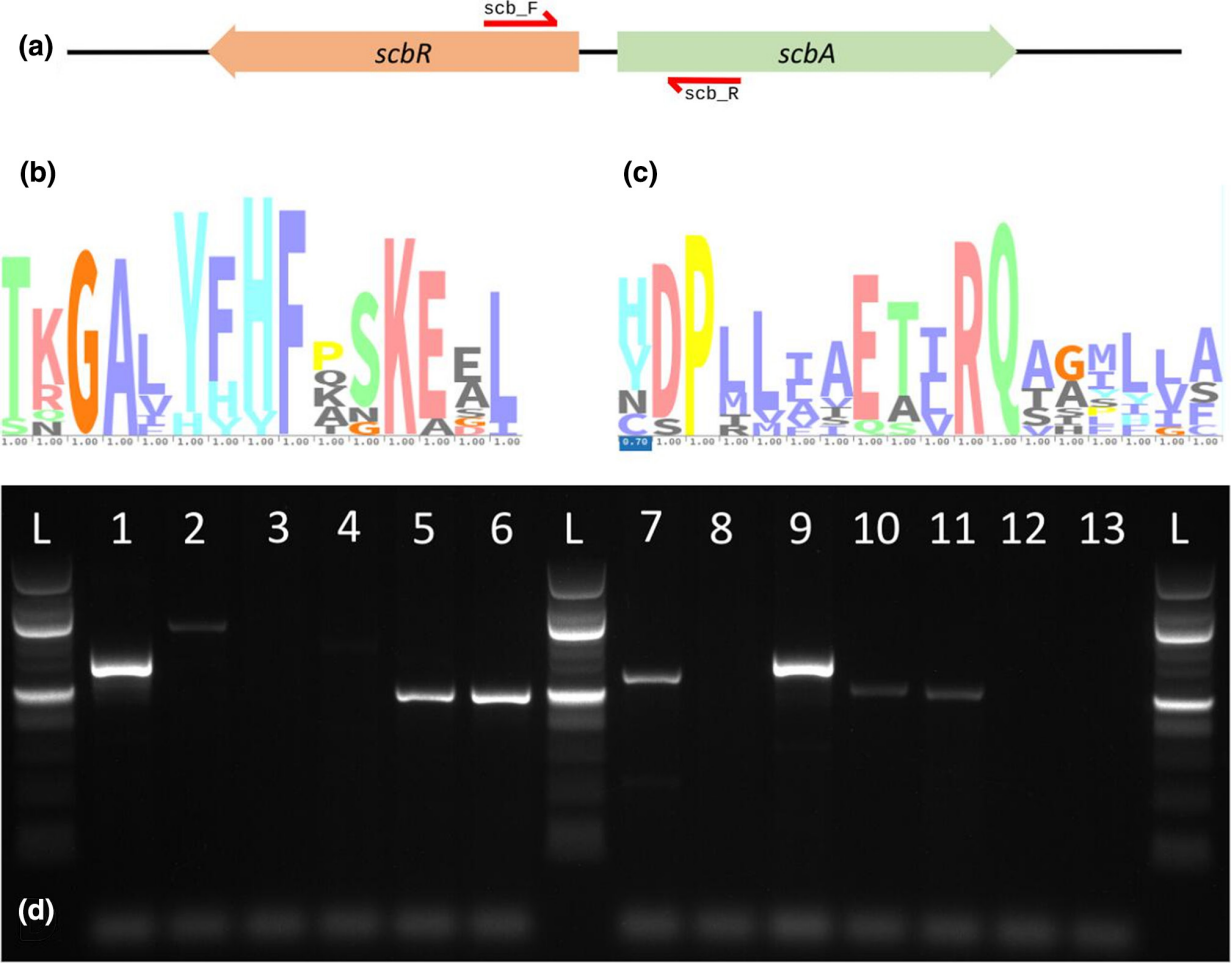
Target genes, target motifs, and PCR results. (**a**) *scbR* and *scbA* genes in divergent orientation, with primer binding sites indicated by red arrows. (**b**) YFHF motif and surrounding amino acids in ScbR homologues, (**c**) ETxRQ motif and surroundings in ScbA homologues. (**d**) Agarose gel electrophoresis (1.7%) of PCR products, see Table 3 for identification of lanes. L=NEB 100 bp ladder.

We first analysed *Streptomyces* genomes to evaluate promising targets. To detect autoregulatory systems, we used the *Streptomyces coelicolor* A3(2) γ-butyrolactone biosynthesis protein ScbA and its receptor protein ScbR to search a custom cblaster database composed of all *Streptomyces* assemblies available in GenBank. A first query with ScbA to detect biosynthesis genes yielded 1558 occurrences of ScbA homologues in 1020 assemblies. Next, we searched for co-occurrences of ScbA and ScbR homologues with a maximum intergenic distance 600 bp, yielding 1092 instances of co-occurrence in 800 assemblies. Since a PCR assay for collocated genes would require one primer in each gene, the orientation of the genes towards each other was crucial. Therefore, the 1092 co-occurrences of ScbA and ScbR homologues were investigated for their orientation towards each other, which showed that the majority (985) of hits showed a divergent (i.e. back-to-back) orientation of the two genes. Therefore, this orientation was chosen for primer design. As the design of degenerate primers relies on conserved motifs, alignments of the protein sequences of divergently aligned homologues were visualized to reveal conserved amino acid residues. From this, two potential primer binding sites could be identified: in ScbR homologues, a strongly conserved YFHF motif in the DNA-binding domain was selected (Fig. 1a, b). In ScbA homologues, in the absence of an equally conserved site, a less conserved ETxRQ motif was chosen as the most promising site (Fig. 1c). Primers were designed according to a *Streptomyces* codon table, resulting in the primers scb_F and scb_R (Table 1).

**Table 1.** Primer sequences

Primer	Target gene	Sequence
scb_F	*scbR*	CCGCTCCTTGCTSGGRAARTGRAARTA
scb_R	*scbA*	GCCGCTCTGGCGVABSGTYTC

### Predicting efficacy of primers

We then compared the frequency of different motifs at the chosen primer binding sites to predict efficiency *in silico*. While the chosen motifs were highly conserved, they were not universally conserved among all sequences. Therefore, the primers would not be able to amplify all sequences, especially if they had mismatches at the 3′ end of the primer. To estimate the likely efficiency of the primers, the composition of the chosen binding sites in the 985 divergently oriented ScbA/ScbR homologes was analysed, with the last amino acid of each motif deemed less important for amplification since it would not constitute the 3′ end of the primer (Table 2). YFHx was present in 93.5 % of ScbR homologues, ETxRx was present in 74.2 % of ScbA homologues and they co-occurred in 69 % of divergently oriented gene pairs. Using co-occurrence of these motifs as a proxy for amplification success, the primer pair might be able to amplify 680 out of 985 (69 %) divergently oriented homologues, or 680 out of 1092 (62.3 %) total co-ocurring ScbA and ScbR homologues.

**Table 2.** Most frequent sequence motifs in the chosen primer binding sites

Target	Motif	Count	Comment
ScbR homologues	All motifs	985	
	YFHF	849	Will prime
	YFHx	921	Will likely prime
	YxHF	865	Might not prime
	xFHF	892	Might not prime
	YHHF	13	Will not prime
	HHHF	0	Will not prime
ScbA homologs	All motifs	985	
	ETxRQ	693	Will likely prime
	ETxRx	731	Will likely prime
	ESxRQ	106	Might not prime

### Primer testing

Finally, we evaluated the primers *in vitro* by conducting PCR. The primers were tested on the extracted DNA of several *Streptomyces* isolates as well as the plasmid C73_787, containing the homologues *mmfL* and *mmfR* derived from the SCP1 plasmid of *Streptomyces coelicolor* A3(2) (Fig. 1d, Table 3). Most of the isolates had a genome sequence associated with them, which allowed a prediction of the amplicon length as well as an analysis of the binding sites. While the plasmid, *S. venezuelae, S. avermitilis, S. coelicolor, S. lividans, S. violaceusniger* and *S. hygroscopicus* showed clear bands in the right size (500–700 basepairs), the three Antarctic isolates MA-3I4, MA-2III1 and MA-0IV2 showed no bands or bands of unexpected size. Investigation of the respective coding sequences showed that they all contained relatively rare amino acid substitutions in the first and/or second N-terminal positions of the conserved primer binding motif (i.e. the 3′ site of the primer), making primer binding unlikely and confirming our predictions (Table 2). However, a larger sample of variants would be necessary to establish generalizable rules about primer efficacy on different variants. Sanger sequencing of bands from *S. violaceusniger* and *S. hygroscopicus *, for which no genome sequence was available, revealed amplification of the correct genes. Furthermore, the genomes of *S. venezuelae* and *S. coelicolor* each contain two potential targets for the primers. However, only one band was observed in each sample, with the amplified variant confirmed by Sanger sequencing. This can be attributed to a bias of the degenerate primers, which preferably amplify one gene variant over another. *S. albus* and *Flavobacterium johnsoniae* were included as true negatives, since their genomes do not contain any *scbR/scbA* homologue pairs.

**Table 3.** PCR results including template DNA, expected band size, observed band size, binding site motifs and notes. Binding site amino acids highlighted in bold if they diverge from the YFHF and ETxRQ motif

No	Sample	Expected (bp)	Observed (bp)	ScbA motif	ScbR motif	Notes
1	Plasmid C73_787 (mmfL/mmfR)	600	600	ETIRQ	YFHF	*mmfL/mmfR* confirmed
2	* Streptomyces* sp. MA-314	516	1000	E**A**VRQ	YFHF	
3	* Streptomyces* sp. MA-2III1	627	none	E**A**IRQ	**HH**HF	
4	* Streptomyces* sp. MA-0IV2	597	none	E**S**VRQ	Y**H**HF	
5	* S. hygroscopicus* AM-3672	n/a	500			*scbA/scbR* homologues confirmed (no genome available)
6	* S. violaceusniger* KCC-S 0850	n/a	500			*scbA/scbR* homologues confirmed (no genome available)
7	* S. venezuelae* ATCC 10712	512/590	550	ETVRQ	YFH**Y**	*sgnL/sgnR* confirmed
8	* S. albus* J1074	none	none			
9	* S. avermitilis* DSM46492	697	700	ETLRQ	YFHF	matching *scbA/scbR* homologues confirmed
10	* S. coelicolor* A3(2)	513/600	500	ETLRQ	YFHF	*scbA/scbR* confirmed
11	*S. lividans* TK24	533	500	ETLRQ	YFHF	matching *scbA/scbR* homologues confirmed
12	* Flavobacterium johnsoniae *	none	none			
13	Water	none	none			

**Fig. 2.**
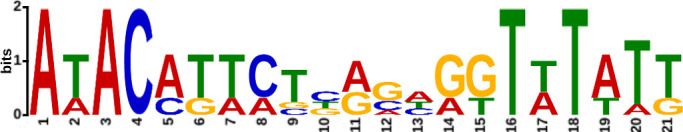
MEME motif logo showing the ARE sequence.

Since the amplification of the intergenic region between *scbA* and *scbR* homologues was confirmed by Sanger sequencing, we wondered whether we could use these sequences to detect the autoregulatory response elements (AREs), the DNA motifs that the cognate receptors and pseudoreceptors bind to. To do this, we ran a MEME motif enrichment analysis on the Sanger sequences obtained from the seven PCR products. This revealed the ARE motif as the most conserved motif among the sequences with an E-value of 4.4E-12 (Fig. 2).

## Discussion

In the present work, we designed a degenerate primer set for the detection of divergently oriented *scbR/scbA* homologues. We predict that this set will be able to amplify 62 % of all co-occurring *scbR/scbA* homologues in *Streptomyces* genomes. The 62 % detection rate could be increased further by designing additional primer sets for different orientations as well as less common motifs. The primer set provides a useful tool for the detection of γ-butyrolactone-like regulator biosynthesis and receptor genes and can be employed e.g. as a screening to prioritise strains before whole-genome sequencing, or as a (meta)genomic library screen. After whole-genome sequencing, the pseudoreceptor can be identified and knocked out, likely leading to de-repression of the regulated biosynthetic gene cluster. Variations of these primers could also be employed for knockouts of *scbA* and *scbR* homologues using CRISPR-Cas9 without WGS, thereby leading to changes in strain metabolite profiles in an approach similar to the one demonstrated by Culp *et al*. [20] Furthermore, we were able to detect the previously described ARE motifs in the intergenic regions between *scbR* and *scbA* homologues. This allows for the identification of the specific ARE for each sample. Since ARE sequences are often highly conserved within a regulatory system, it could be feasible to use CRISPR-Cas9 to introduce mutations in the AREs, thereby potentially inducing secondary metabolite production without the necessity for whole-genome sequencing.
